# How psychopathy is associated with the level and change of impulsivity in correctional treatment

**DOI:** 10.1192/j.eurpsy.2024.343

**Published:** 2024-08-27

**Authors:** A. Voulgaris, P. Briken, E. Stück

**Affiliations:** Institute for Sex Research, Sexual Medicine and Forensic Psychiatry, Medical University Hamburg, Hamburg, Germany

## Abstract

**Introduction:**

Research indicates that psychopathy can hinder treatment success and can lead to dropout. Impulsivity is a complex construct that overlaps with psychopathic personality traits and is often targeted in forensic psychotherapy due to its relation to the risk of reoffending.

**Objectives:**

Our aim was to investigate the overlap between psychopathy and impulsivity and the influence of psychopathic traits on change in impulsivity.

**Methods:**

We conducted a pre-post-study for measures of psychopathy and impulsivity in men imprisoned for sexual and non-sexual violent offenses. All participants took part in standardized pre- and post-treatment ratings shortly after admission as well as after an average of 19 months (n=370 for pre-rating, n=168 for post-rating). Psychopathy was measured via the PCL-R, impulsivity with the BIS-15.

We calculated two-tailed Pearson correlations for BIS-15 Pre-, Post-, and Change Scores and the PCL-R. In a second step, the BIS-15 pre-post-differences were compared using independent t-Tests, effect sizes were calculated using Cohen’s d (small, medium, and large effect sizes are d = .20, .50, and .80). Further, unpaired t-tests were carried out to compare between participants with lower and higher PCL-R sum scores (median split, mdn= 15.8, M=15.5, SD=7.9).

**Results:**

In the total population a significant reduction of self assessed impuslivity can be demonstrated for total impulsivity (p<.001, cohens d= .34) nonplanned (p<.001, cohens d= .39) and motor impulsivity (p=.004, cohens d= .23). In both groups, with higher and lower psychopathic traits, a significant reduction in total  and nonplannend impulsivity can be seen.

While the reduction in total impulsivity was 0.9 points higher in the group with higher psychopathy, the difference was not significant, *t*(147.8)= -1.1, *p* = .285. Also, the nonplanned impulsivity was showed a stronger reduction in the high PCL group, though the effect was not significant, t, *t*(166)= -1.2, *p* = .243.

**Table 1.** Correlationen between BIS-15 post-ratings and PCL-R

		PCL-R				
		PCL-R Sum	Interpersonal	affective	lifestyle	antisocial
BIS-15 prä (n=370)	Total Impulsivity	.20 **	-.14 **	.02	.34 **	.28 **
BIS-15 post (n=168)	Total Impulsivity	.33**	.04	.20**	.35**	.36**
BIS-15 Change (n=168)	Total Impulsivity	-.03	-.15	-.09	.07	.01

Note: * correlation significant für p ≤ .05; ** correlation significant für p ≤ .001.

**Conclusions:**

We demonstrate a significant correlation between psychopathy and impulsivity, especially regarding facets 3 and 4, but also for the sum score. Neither the PCL-R sum core, nor the facets correlate with the change in impulsivity during treatment progress in the STU. In both groups, with higher and lower psychopathy, impulsivity was reduced during therapy but there was no significant difference in the change scores. Our results underline that treatment progress can be achieved also in patients with higher psychopathic traits.

**Disclosure of Interest:**

None Declared

